# A three-dimensional whole-body model to predict human walking on level ground

**DOI:** 10.1007/s10237-022-01629-7

**Published:** 2022-10-26

**Authors:** Dan Hu, David Howard, Lei Ren

**Affiliations:** 1 School of Mechanical Aerospace and Automotive Engineering, Coventry University, Coventry, UK; 2 School of Science, Engineering and Environment, Univesity of Salford, Salford, UK; 3 School of Mechanical Aerospace and Civil Engineering, University of Manchester, Manchester, UK

**Keywords:** Locomotion, Inverse dynamics, Optimization, Predictive models, Three-dimensional

## Abstract

Predictive simulation of human walking has great potential in clinical motion analysis and rehabilitation engineering assessment, but large computational cost and reliance on measurement data to provide initial guess have limited its wide use. We developed a computationally efficient model combining optimization and inverse dynamics to predict three-dimensional whole-body motions and forces during human walking without relying on measurement data. Using the model, we explored two different optimization objectives, mechanical energy expenditure and the time integral of normalized joint torque. Of the two criteria, the sum of the time integrals of the normalized joint torques produced a more realistic walking gait. The reason for this difference is that most of the mechanical energy expenditure is in the sagittal plane (based on measurement data) and this leads to difficulty in prediction in the other two planes. We conclude that mechanical energy may only account for part of the complex performance criteria driving human walking in three dimensions.

**Supplementary Information:**

The online version contains supplementary material available at 10.1007/s10237-022-01629-7.

## Introduction

Walking is a motor task requiring sophisticated coordination of multiple body segments and joints. A powerful approach to understand the biomechanics and control strategies underpinning human walking is the use of predictive simulation that calculates movements based on a mathematical description of the neuro-musculoskeletal system (Chow and Jacobson 1971; Davy and Audu 1987; Yamaguchi 1990; Koopman et al. 1995; Anderson and Pandy 2001). Predictive simulation using complex forward dynamics musculoskeletal models, driven by the major muscles actuating the joints of the lower limbs, has achieved good predictions of body movements and motor control processes in gait (Anderson and Pandy 2001; Dorn et al. 2015; Shourijeh and McPhee 2014; Sreenivasa et al. 2017). This approach has been widely studied possibly because it coincides with the natural sequence of neuromuscular control (Zajac and Winters 1990). However, the solution of a large number of differential equations leads to expensive computation. Nowadays the use of direct collocation method and faster solvers (e.g., SNOPT and IPOPT) has sped up the computation of optimization based on forward dynamics, but the initial guess or part of the initial guess (called quasi-random initial guess) is required from walking data (Lee and Umberger 2016; Falisse et al. 2019). On the contrary, predictive simulation using inverse dynamics driven by kinematics (such as joint motions) has the advantage of computational efficiency and experimental independence. And it is more straightforward to impose the kinematic constraints that define walking in inverse dynamic model. Research that applied inverse dynamics in predictive simulation are less common but existing work have shown fast calculation speed (512CPU seconds on a Pentium (R) 4, 3.46 GHz computer, Xiang et al. 2009).

In the studies that have applied inverse dynamics in predictive simulation, some work focused on the lower limbs motion prediction (Martin and Schmiedeler 2014) and others simplified upper body (trunk, arms and head) as one part (Yen and Nagurka 1987; Ren et al. 2007). These studies either predicted the gait during the single stance phase only (Yen and Nagurka 1987) or in the sagittal plane only (Saidouni and Bessonnet 2003; Martin and Schmiedeler 2014). Although three-dimensional whole-body walking prediction has been implemented (Fregly et al. 2007; Kim et al. 2008; Xiang et al. 2009; Bessonnet et al. 2010), the foot–ground interface was modelled using springs and dampers (Fregly et al. 2007) or predefined polygon points (Kim et al. 2008; Xiang et al. 2009; Bessonnet et al. 2010). These unnatural constraints are likely to adversely affect the quality of the gait prediction.

Considering the optimal criterion that represents the motor task objective during walking, various criteria have been studied (Marshall et al. 1989; Kai et al. 2012). Experimental observations have shown minimal energy cost per unit distance is achieved at selected walking frequency and stride length (Cavagna and Kaneko 1977; Miller et al. 2012). Based on this observation, energy-based performance criteria, such as mechanical or metabolic energy expenditure, have been applied to predict human walking (Anderson and Pandy 2001; Ren et al. 2007). However, criteria based on muscle effort and muscle fatigue have also shown promise (Ackermann and van de Bogert 2010; Miller et al. 2012), with some concluding that it predicted more realistic movements comparing to energy-based criteria (Ackermann and van de Bogert 2010). A fact is that metabolic energy or muscle effort-based criteria require the modelling of individual muscles, including solving for muscle redundancy, which will rise the computational expense. Another optimal criterion is the time integrals of the normalized joint torques that can be applied without modelling muscles (Koopman et al. 1995; Xiang et al. 2009).

Therefore, our aim was to construct a three-dimensional whole-body predictive model of walking. We included all the joints of a human body (ankle, knee, hip, waist, shoulder, elbow and neck) and imposed no artificial constraints on body movement. We aimed to apply different performance criteria and identify the criterion that produces more accurate predictions: mechanical energy expenditure and the sum of the time integrals of the normalized joint torques. The model was established by building upon our previous work of a two-dimensional model, which achieved promising predictions of human walking in the sagittal plane (Ren et al. 2007). Extending it to a three-dimensional predictive model has potential applications in areas such as walking balance study, three-dimensional clinical motion analysis and three-dimensional rehabilitation engineering assessment.

## Method

### The multi-segment model

**Fig. 1:**
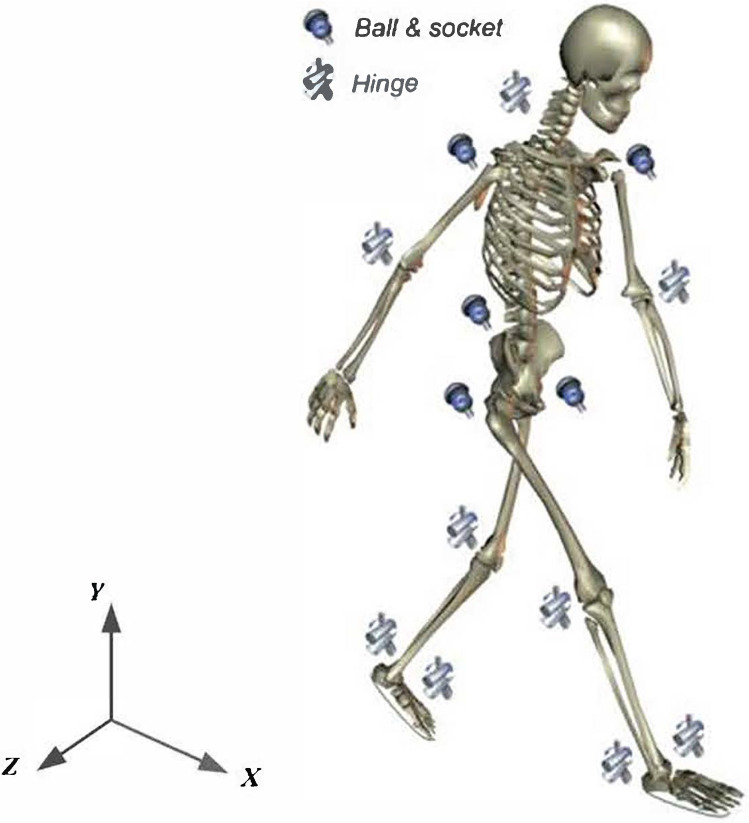
The three-dimensional whole-body skeletal model with 13 segments and 12 connecting joints. The *X* axis of the global coordinate system points in the progression direction, the *Y* axis points vertically upwards, and the *Z* axis points in the lateral direction according to the right-hand rule

Referring to Fig. 1, the human body is modelled as a three-dimensional multi-segment articulated system, with 13 segments and 12 joints. There are 13 segments including the head, torso, pelvis, upper arms, forearms, thighs, shanks, and feet. These segments are connected by the joints of the neck, waist, shoulder, elbow, hip, knee, and ankle. The neck, elbow and knee joints are modelled as simple hinge joints, each with one degree of freedom (DoF). The waist, shoulder and hip joints are modelled as ball and socket joints, each with three DoFs. The ankle joint is modelled as a universal joint, representing two anatomical joints (the Subtalar and Talocrural joints), with the two joint axes intersecting at the ankle joint centre. Therefore, the model has a total of 24 DoFs. Anthropometric data for each body segment, including segment mass, centre of mass positions, and moment of inertia, are based on the data of de Leva (Table 1) (de Leva 1996).

### Kinematics

Instead of using direction cosines or Euler angles to represent each joint rotation in three dimensions, we employed Euler parameters (also known as a unit quaternion). Compared to Euler angles, using quaternions avoids the problem of gimbal lock. Furthermore, the use of quaternion multiplication to map between coordinate systems has the advantage of less computational cost than matrix multiplication (Goldman 2009). If unit quaternion $\Lambda$ is used to describe the orientation from coordinate system 1 to 2 ($\Lambda^{^{\prime}}$ is the conjugate quaternion), $\mathop{x}\limits^{\rightharpoonup} _{2} = \Lambda \otimes \mathop{x}\limits^{\rightharpoonup} _{1} \otimes \Lambda ^{\prime}$ can be used to map $\overrightarrow {x}$ from system 1 to system 2. Here the unit quaternion is calculated by $\Lambda = [\lambda_{0} ,\lambda_{1} ,\lambda_{2} ,\lambda_{3} ]$, $\lambda_{0} = \cos (\theta /2)$, $\lambda_{i} = u_{i} \sin (\theta /2),\;i = 1,2,3$, where $\theta$ is the joint rotation angle and $\overrightarrow {u} = [u_{1} ,u_{2} ,u_{3} ]$ is the direction of joint axis. The angular velocity $\omega$ and angular acceleration $\alpha$ can be obtained using the simple linear calculations $\mathop{\omega }\limits^{\rightharpoonup} = 2L\dot{\Lambda }$ and $\mathop{\alpha }\limits^{\rightharpoonup} = 2L\ddot{\Lambda }$, where $\dot{\Lambda } = [\dot{\lambda }_{0} ,\;\dot{\lambda }_{1} ,\;\dot{\lambda }_{2} ,\;\dot{\lambda }_{3} ]$, $\ddot{\Lambda } = [\ddot{\lambda }_{0} ,\ddot{\lambda }_{1} ,\ddot{\lambda }_{2} ,\ddot{\lambda }_{3} ]$, and $$L = \left[ {\begin{array}{*{20}c} { - \lambda_{1} } & {\lambda_{0} } & {\lambda_{3} } & { - \lambda_{2} } \\ { - \lambda_{2} } & { - \lambda_{3} } & {\lambda_{0} } & {\lambda_{1} } \\ { - \lambda_{3} } & {\lambda_{2} } & { - \lambda_{1} } & {\lambda_{0} } \\ \end{array} } \right]$$.

**Fig. 2:**
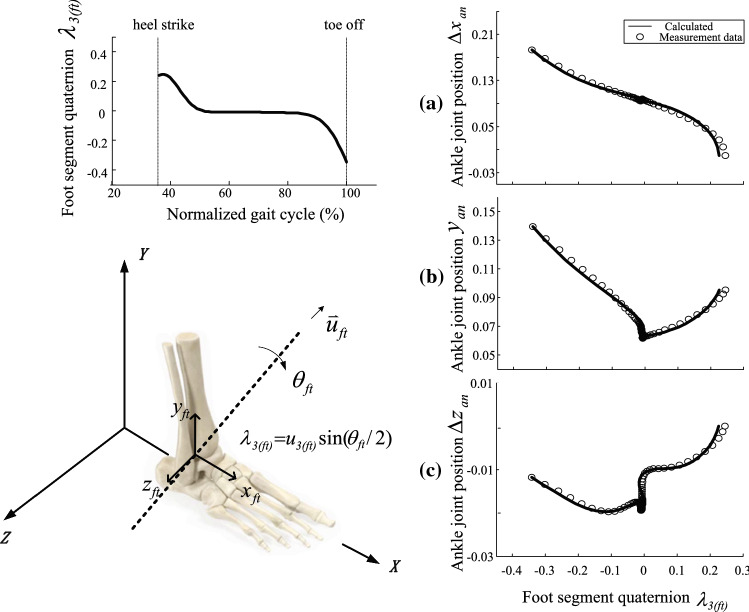
Three-dimensional ankle–foot kinematics during foot rollover in the stance phase. The Euler parameters representing the foot segment orientation are derived from the rotation angle $\theta_{{{\text{ft}}}}$ and rotation axis $\mathop{u}\limits^{\rightharpoonup} = [u_{{1({\text{ft}})}} ,u_{{2({\text{ft}})}} ,u_{{3({\text{ft}})}} ]$. As the angular motion of the foot is modelled as a 1 DoF rotation, the foot kinematics can be described as functions of quaternion element $\lambda_{{3({\text{ft}})}} = u_{{\text{3(ft)}}} \sin (\theta_{{{\text{ft}}}} /2)$. Top left is the time trajectory of quaternion element $\lambda_{{3({\text{ft}})}}$ for stance foot rotation from heel strike to toe off, i.e. from 36 to 100% of the gait cycle. On the right is the mathematical representation of the ankle–foot kinematics during stance compared with measurement data (circles): **a** *x* coordinate of ankle joint position (relative to heel strike moment), unit: m; **b** y coordinate of ankle joint position, unit: m; and **c** *z* coordinate of ankle joint position (relative to heel strike moment), unit: m. The subject (age: 25; weight: 68.8 kg; height: 177 cm) walked at 1.3806 ms^−1^, and the cycle period was 1.08 s

The stance foot is modelled as a rigid body rolling on the ground without slipping (Fig. 2). Building on our previous work on foot kinematics (Ren et al. 2007), the three-dimensional foot kinematics during the stance phase are described by

1 $$\left\{ \begin{gathered} \Delta x_{{{\text{an}}}} = f(\lambda_{{3({\text{ft}})}} ) \hfill \\ y_{{{\text{an}}}} = g(\lambda_{{3({\text{ft}})}} ) \hfill \\ \Delta z_{{{\text{an}}}} = h(\lambda_{{3({\text{ft}})}} ) \hfill \\ \end{gathered} \right.$$

where: $\Delta x_{{{\text{an}}}} = x_{{{\text{an}}}} - x_{{{\text{an}}}}^{{\text{(hs)}}}$; $\Delta z_{{{\text{an}}}} = z_{an} - z_{{{\text{an}}}}^{{\text{(hs)}}}$; $x_{{{\text{an}}}}$, $y_{{{\text{an}}}}$ and $z_{{{\text{an}}}}$ are the $x$, $y$ and $z$ coordinates of the ankle joint centre; $x_{{{\text{an}}}}^{{({\text{hs}})}}$ and $z_{{{\text{an}}}}^{{({\text{hs}})}}$ are the $x$ and $z$ coordinates of the ankle joint centre at the moment of heel strike; $\lambda_{{3({\text{ft}})}}$ is an element of the unit quaternion $\Lambda_{{{\text{ft}}}}$. Figure 2 depicts the output of the foot model with respect to foot rotation when the plantar roll over shape is described by cubic spline interpolation knots. Based on Eq. 1, the accelerations of the ankle joint centre in three dimensions $[\ddot{x}_{\text{an}} ,\ddot{y}_{\text{an}} ,\ddot{z}_{\text{an}} ]$ can be achieved by differentiating it twice.

As there is at least one foot contacting the ground throughout the walking cycle, the calculation sequence of the multi-body system starts from the stance foot. Thus, the coordinates of the joint centres in the multi-segment model are derived sequentially, starting at the ankle joint centre of the stance leg:

2 $$\left\{ \begin{gathered} \overset{\lower0.5em\hbox{$\smash{\scriptscriptstyle\rightharpoonup}$}} {r} _{1} = [x_{{{\text{an}}}} ,y_{{{\text{an}}}} ,z_{{{\text{an}}}} ]\begin{array}{*{20}l} {\begin{array}{*{20}l} {} & {} \\ \end{array} } & {\begin{array}{*{20}c} {} & {} \\ \end{array} } \\ \end{array} \hfill \\ \overset{\lower0.5em\hbox{$\smash{\scriptscriptstyle\rightharpoonup}$}} {r} _{{i + 1}} = \overset{\lower0.5em\hbox{$\smash{\scriptscriptstyle\rightharpoonup}$}} {r} _{i} + \begin{array}{*{20}c} {\Lambda _{i}^{g} \otimes P_{i}^{{i + 1}} \otimes \Lambda _{i}^{{g}{\prime}}} & {i = 1,2,3,4...} \\ \end{array} \hfill \\ \end{gathered} \right.$$

Here, $\mathop{r}\limits^{\rightharpoonup} _{i}$ is the position vector of the $i$ th joint centre. The $i$ th joint connects the $i$ th and $i + 1$ th segments. $\Lambda_{i}^{g}$ is a unit quaternion indicating the orientation of the $i$ th segment in the global system, and it is computed by $\Lambda_{i}^{g} = \Lambda_{i - 1} \otimes \Lambda_{i - 2} \cdots \otimes \Lambda_{ft}$, where quaternion $\Lambda_{i}$ represents the $i$ th joint rotation. $\Lambda_{i}^{g}{^{\prime}}$ is the conjugate quaternion of $\Lambda_{i}^{g}$. In Eq. 2, $P_{i}^{i + 1}$ indicates the position vector in the $i$ th segment pointing from the $i$ th joint centre to the $i + 1$ th joint centre. Differentiating Eq. 2 twice, the accelerations of the joint centre are given by:

3 $$\left\{ \begin{gathered} \ddot{\overset{\lower0.5em\hbox{$\smash{\scriptscriptstyle\rightharpoonup}$}} {r} }_{1} = \left[ {\ddot{x}_{{{\text{an}}}} ,\ddot{y}_{{{\text{an}}}} ,\ddot{z}_{{{\text{an}}}} } \right]\begin{array}{*{20}c} {} & {} & {} & {\begin{array}{*{20}c} {} & {} & {\begin{array}{*{20}c} {} & {} & {} & {\begin{array}{*{20}c} {} & {} & {} & {\begin{array}{*{20}c} {} & {\begin{array}{*{20}c} {} & {} \\ \end{array} } & {} \\ \end{array} } \\ \end{array} } \\ \end{array} } \\ \end{array} } \\ \end{array} \hfill \\ \ddot{\overset{\lower0.5em\hbox{$\smash{\scriptscriptstyle\rightharpoonup}$}} {r} }_{{i + 1}} = \ddot{\overset{\lower0.5em\hbox{$\smash{\scriptscriptstyle\rightharpoonup}$}} {r} }_{i} + \ddot{\Lambda }_{i}^{g} \otimes P_{i}^{{i + 1}} \otimes \Lambda _{i}^{g\prime} + \Lambda _{i}^{g} \otimes P_{i}^{{i + 1}} \otimes \ddot{\Lambda }_{i}^{g\prime} + 2\dot{\Lambda }_{i}^{g} \otimes P_{i}^{{i + 1}} \otimes \dot{\Lambda }_{i}^{g\prime} \begin{array}{*{20}c} {} & {i = 1,2,3,4...} \\ \end{array} \hfill \\ \end{gathered} \right.$$

So given the quaternion of each joint rotation, Eqs. 1–3 can be used to calculate the coordinates of the joint centre positions and their accelerations. Thereafter, the positions and accelerations of the mass centre of each segment are derived based on anthropometric data.

### Kinetics

The inverse dynamics method was used to calculate the joint kinetics, external forces and external moments in walking. The dynamics of the $i$ th body segment is determined as follows (Siegler and Liu 1997; Winter 2005):

4 $$\left\{ \begin{gathered} m_{i} \ddot{\overset{\lower0.5em\hbox{$\smash{\scriptscriptstyle\rightharpoonup}$}} {r} }_{{ci}} = m_{i} \overset{\lower0.5em\hbox{$\smash{\scriptscriptstyle\rightharpoonup}$}} {g} + \sum\limits_{{k = 1}}^{{n_{{ji}} }} {\overset{\lower0.5em\hbox{$\smash{\scriptscriptstyle\rightharpoonup}$}} {F} _{{jk}}^{{(i)}} + } \sum\limits_{{k = 1}}^{{n_{{ei}} }} {\overset{\lower0.5em\hbox{$\smash{\scriptscriptstyle\rightharpoonup}$}} {F} _{{ek}}^{{(i)}} } \hfill \\ I_{{ci}} \cdot \overset{\lower0.5em\hbox{$\smash{\scriptscriptstyle\rightharpoonup}$}} {\alpha } _{i} + \overset{\lower0.5em\hbox{$\smash{\scriptscriptstyle\rightharpoonup}$}} {\omega } _{i} \times (I_{{ci}} \cdot \overset{\lower0.5em\hbox{$\smash{\scriptscriptstyle\rightharpoonup}$}} {\omega } _{i} ) = \sum\limits_{{k = 1}}^{{n_{{ji}} }} {\overset{\lower0.5em\hbox{$\smash{\scriptscriptstyle\rightharpoonup}$}} {M} _{{jk}}^{{(i)}} + \sum\limits_{{k = 1}}^{{n_{{ei}} }} {\overset{\lower0.5em\hbox{$\smash{\scriptscriptstyle\rightharpoonup}$}} {M} _{{ek}}^{{(i)}} + } } \sum\limits_{{k = 1}}^{{n_{{ji}} }} {(\overset{\lower0.5em\hbox{$\smash{\scriptscriptstyle\rightharpoonup}$}} {r} _{{jk}}^{{(i)}} \times \overset{\lower0.5em\hbox{$\smash{\scriptscriptstyle\rightharpoonup}$}} {F} _{{jk}}^{{(i)}} ) + } \sum\limits_{{k = 1}}^{{n_{{ei}} }} {(\overset{\lower0.5em\hbox{$\smash{\scriptscriptstyle\rightharpoonup}$}} {r} _{{ek}}^{{(i)}} \times \overset{\lower0.5em\hbox{$\smash{\scriptscriptstyle\rightharpoonup}$}} {F} _{{ek}}^{{(i)}} )} \hfill \\ \end{gathered} \right.$$

Therefore, the sums of the external forces and moments (ground reactions) acting on the foot are thereby obtained by adding the motion equations of all body segments together:

5 $$\left\{ \begin{gathered} \overset{\lower0.5em\hbox{$\smash{\scriptscriptstyle\rightharpoonup}$}} {F} _{{{\text{gr}}}} + \overset{\lower0.5em\hbox{$\smash{\scriptscriptstyle\rightharpoonup}$}} {F} _{{{\text{gl}}}} = \sum\limits_{{i - 1}}^{n} {[m_{i} (\ddot{\overset{\lower0.5em\hbox{$\smash{\scriptscriptstyle\rightharpoonup}$}} {r} }_{{ci}} - g)]} \hfill \\ \overset{\lower0.5em\hbox{$\smash{\scriptscriptstyle\rightharpoonup}$}} {M} _{{{\text{gr}}}} + \overset{\lower0.5em\hbox{$\smash{\scriptscriptstyle\rightharpoonup}$}} {M} _{{{\text{gl}}}} = \sum\limits_{{i = 1}}^{n} {[I_{{ci}} } \cdot \overset{\lower0.5em\hbox{$\smash{\scriptscriptstyle\rightharpoonup}$}} {\alpha } _{i} + \overset{\lower0.5em\hbox{$\smash{\scriptscriptstyle\rightharpoonup}$}} {\omega } _{i} \times (I_{{ci}} \cdot \overset{\lower0.5em\hbox{$\smash{\scriptscriptstyle\rightharpoonup}$}} {\omega } _{i} ) - \sum\limits_{{i = 1}}^{n} {\sum\limits_{{k = 1}}^{{n_{{ei}} }} {(\overset{\lower0.5em\hbox{$\smash{\scriptscriptstyle\rightharpoonup}$}} {r} _{{ek}}^{{(i)}} \times \overset{\lower0.5em\hbox{$\smash{\scriptscriptstyle\rightharpoonup}$}} {F} _{{ek}}^{{(i)}} )} } - \sum\limits_{{i = 1}}^{n} {\sum\limits_{{k = 1}}^{{n_{{ji}} }} {(\overset{\lower0.5em\hbox{$\smash{\scriptscriptstyle\rightharpoonup}$}} {r} _{{jk}}^{{(i)}} \times \overset{\lower0.5em\hbox{$\smash{\scriptscriptstyle\rightharpoonup}$}} {F} _{{jk}}^{{(i)}} )} } \hfill \\ \end{gathered} \right.$$

In Eq. 4: $$m_{i}$$ is the mass of the $$i$$ th segment; $$I_{ci}$$ is the moment of inertia of the $$i$$ th segment; $$\ddot{\mathop{r}\limits^{\rightharpoonup} }_{ci}$$ is the translational acceleration vector of the $$i$$ th segment center of mass; $$\overset{\lower0.5em\hbox{$\smash{\scriptscriptstyle\rightharpoonup}$}} {r} _{{{\text{jk}}}}^{{(i)}}$$ is the position vector of the $$k$$ th joint force from the mass center of the $$i$$ th segment; $$\overset{\lower0.5em\hbox{$\smash{\scriptscriptstyle\rightharpoonup}$}} {r} _{{{\text{ek}}}}^{{(i)}}$$ is the position vector of the $$k$$ th external force from the mass center of the $$i$$ th segment; $$\overset{\lower0.5em\hbox{$\smash{\scriptscriptstyle\rightharpoonup}$}} {F} _{{{\text{jk}}}}^{{(i)}}$$ is the $$k$$ th joint force vector acting on the $$i$$ th segment; $$\overset{\lower0.5em\hbox{$\smash{\scriptscriptstyle\rightharpoonup}$}} {F} _{{{\text{ek}}}}^{{(i)}}$$ is the $$k$$ th external force vector acting on the $$i$$ th segment; $$\overset{\lower0.5em\hbox{$\smash{\scriptscriptstyle\rightharpoonup}$}} {M} _{{{\text{jk}}}}^{{(i)}}$$ is the $$k$$ th net muscle moment acting on the $$i$$ th segment; $$\overset{\lower0.5em\hbox{$\smash{\scriptscriptstyle\rightharpoonup}$}} {M} _{{{\text{ek}}}}^{{(i)}}$$ is the $$k$$ th external moment acting on the $$i$$ th segment. In Eq. 5: $$\mathop{F}\limits^{\rightharpoonup} _{gr} ,\mathop{M}\limits^{\rightharpoonup} _{gr}$$ are the ground force and moment vectors acting on the right foot; $$\mathop{F}\limits^{\rightharpoonup} _{gl} ,\mathop{M}\limits^{\rightharpoonup} _{gl}$$ are the ground force and moment vectors acting on the left foot; $$n$$ is the total number of segments; $$n_{ji}$$ is the number of joint forces acting on $$i$$ th segment; and $$n_{ei}$$ is the number of external forces acting on $$i$$ th segment.

Therefore, the ground force and moment acting on the stance foot can be obtained directly from Eqs. 4–5 in the single stance phase. However, during double stance phase, the problem of determining the ground reactions under each foot becomes indeterminate.

### Transition assumption

In order to solve the indeterminate problem, we applied a transition assumption function that closely fits the measured ground reactions. This function combined the linear transfer assumption (Ren et al. 2007) and the smooth transition assumption (STA) (Ren et al. 2008) from previous work.

Analytical functions in exponential form were found to match the experiment data well, with the function values (ground forces and moments on the trailing foot) changing smoothly towards zero. Specifically, the transition functions for the ground reaction components $F_{y}$ and $M_{z}$ of the trailing foot are given by:

6 $$\left\{ \begin{gathered} \frac{{F_{y} }}{{F_{yo} }} = e^{{ - (t/T_{{{\text{ds}}}} )^{3} }} \hfill \\ \frac{{M_{z} }}{{M_{zo} }} = e^{{ - (t/T_{{{\text{ds}}}} )^{3} }} \hfill \\ \end{gathered} \right.$$

where: $T_{{{\text{ds}}}}$ is half the double support duration; $F_{yo}$ is the vertical force at contralateral heel strike; and $M_{zo}$ is the sagittal plane moment at contralateral heel strike. The longitudinal and lateral forces, as well as the reaction moment in frontal and transversal plane are obtained based on the linear assumption, as follows:

7 $$F_{x} = \mu_{x} F_{y}, \; F_{z} = \mu_{z} F_{y}$$

8 $$M_{x} = \varpi_{x} M_{z}, \; M_{y} = \varpi_{y} M_{z}$$

where $\mu_{x}$, ${\mu }_{z}$ are the friction coefficients between horizontal forces $F_{x}$, $F_{z}$ and vertical force $F_{y}$ and $\varpi_{x}$, $\varpi_{y}$ are the transfer ratios between $M_{x}$, $M_{y}$ and $M_{z}$.

**Fig. 3:**
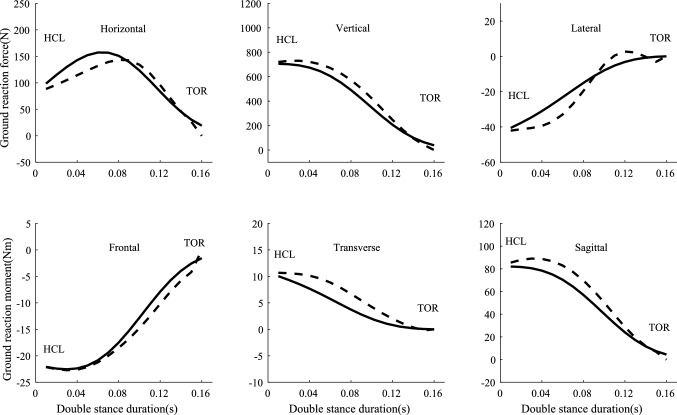
Representative calculated ground forces and moments (dashed line) based on the transition assumption model compared with force plate data (solid line) in the double stance phase from left heel strike to right toe off. The subject walked at 1.3806 ms^−1^

Figure 3 showed the calculated results using the transition assumption model compared with force plate data from a representative subject during normal walking. The figure indicates that the model is in good agreement with the ground reaction measurements. The same subject data was used to validate transition assumption model and to compare with predictive results later. We applied smooth transition to $F_{y}$ (ground reaction force in vertical direction) and $M_{z}$ (ground reaction moment in sagittal plane) due to its good fitting effect from previous research (Ren et al. 2008). The STA assumption has been validated against force plate data for three subjects walking at both normal and fast speed (Ren et al. 2008). Sensitivity analysis about the body segment parameters on the transition function was already conducted in previous research work (Ren et al. 2008). For the transition along the other two directions, we used linear transfer assumptions instead of smooth transition assumptions. We found that relating the ground reaction forces in x and z directions with $F_{y}$ and relating the ground reaction moments in *x* and *y* directions with $M_{z}$ benefitted the convergence of the optimization algorithm.

During walking, the simulated ground reaction forces and moment on each foot are calculated from Eq. 5 and the improved transfer relationships. Then, the resultant force and net muscle moment at each joint are calculated using Eq. 4, starting from the stance foot and working up, segment by segment.

**Table 1:** Anthropometric data

Segment	Mass(kg)	Moment Inertia along *x* axis (kg m^2^)	Moment Inertial along *y* axis (kg m^2^)	Moment Inertial along *z* axis (kg m^2^)
Head	4.7886	0.0223	0.0165	0.0241
Torso	22.2801	0.5817	0.2778	0.4297
Pelvis	7.7073	0.0554	0.0505	0.0445
Humerus	1.8699	0.0184	0.0057	0.0164
Forearm	1.5387	0.0229	0.0018	0.0219
Thigh	9.7704	0.1664	0.0341	0.1664
Shank	2.9877	0.0344	0.0057	0.033
Foot	0.9453	0.0011	0.0003	0.0006

Table 1 shows the anthropometric data used in the whole human body

### Optimization problem

In the optimization process, joint trajectories are the unknowns to be obtained. In order to represent these variables, a set of fifth order Fourier series is used as follows:

9 $$\lambda_{i} = a_{0}^{(i)} + \sum\limits_{k = 1}^{5} {(a_{k}^{(i)} \cos (k\omega t) + b_{k}^{(i)} \sin (k\omega t))}$$

where $\omega = 2\pi /T$ is the angular frequency, $T$ is the walking cycle period. A fifth-order Fourier series was chosen because the signal power of the measurement data is predominantly made up of frequencies below 6 Hz (Winter 2005). Each Fourier series has 11 coefficients, therefore, the total number of unknown parameters amounts to 165 (11 coefficients multiplied by 15 DoFs due to the symmetry of walking gait).

Various performance objectives have been studied and applied in human movement prediction (Marshall et al. 1989; Kai et al. 2012). Mechanical energy expenditure as a criterion has shown promising results with a two-dimensional model (Ren et al. 2007), but its application with a three-dimensional model has not been reported. On the other hand, the sum of the time integrals of the normalized joint torques has been used in predictive simulations (Koopman et al. 1995; Xiang et al. 2009). Here we explored these two criteria in our model.

The mechanical energy during the walking cycle is defined as:

10 $$C_{{{\text{en}}}} (\lambda ) = \int_{0}^{T} {\sum\limits_{i = 1}^{N} {\sum\limits_{k = 1}^{3} {\alpha_{ji}^{(k)} \left| {M_{ji}^{(k)} (\omega_{pi}^{(k)} - \omega_{di}^{(k)} )} \right|} } {\text{d}}t}$$

where: $k$ denotes the coordinate number; $M_{ji}^{(k)}$ is the net muscle moment acting at the $i$ th joint about the $k$ th coordinate axis; $\omega_{pi}^{(k)}$ and $\omega_{di}^{(k)}$ are the $k$ th components of the angular velocities of the $i$ th joint’s proximal and distal segments, respectively; $\alpha_{ji}^{(k)}$ is “1” when joint rotation about the $k$ th coordinate axis is allowed and is “0” when there is no rotation about that axis; and $N$ is the number of joints.

The sum of the time integrals of the absolute normalized joint torques is given by (Koopman et al. 1995):

11 $$C_{{{\text{tor}}}} (\lambda ) = \sum\limits_{i = 1}^{N} {\sum\limits_{k = 1}^{3} {\int_{t = 0}^{T} {\alpha_{ji}^{(k)} \left| {\frac{{M_{ji}^{(k)} }}{{M_{\max ,ji}^{(k)} }}} \right|{\text{d}}t} } }$$

where: $M_{\max ,ji}^{(k)}$ is the maximum absolute value of isometric muscle moment about the $k$ th coordinate axis obtained from the literature (Kumar 1996, Kaminski et al. 1999, Martins et al. 2017, and Gonosova et al. 2018).

The optimization constraints associated with walking and anatomical limitations are as follows:

1. Joint motion constraints:
  $$\lambda_{\min ,i} \le \lambda_{i} \le \lambda_{\max ,i}$$

Since the quaterion *λ* is calculated by joint rotation angle θ, constraining the quaternion value is to constrain the joint rotation angle. Most of the joints rotation angles were constrained in the motion range that the human body could maximally achieve. Waist joints were constrained in a smaller motion range to keep the upper body upright. Neck joint was also constrained in a smaller motion range to ensure the eyes looking forward.

(2) Segment motion constraints:

$\lambda_{\min ,i}^{g} \le \lambda_{i}^{g} \le \lambda_{\max ,i}^{g}$, where $\lambda_{i}^{g}$ is the quaternion of the $i$ th segment in the global coordinate system.

The global quaternion *λ*^*g*^ represents the rotation of the segment in the global coordinate system. In our model, only the torso segment rotation in the global coordinates was constrained. The reason is also to keep the upper body upright.

(3) Kinematic constraints:

$y_{{{\text{tip}}}} (t) > 0$ for a swing foot and $y_{{{\text{tip}}}} (t) = 0$ for a stance foot, where $y_{{{\text{tip}}}}$ is the vertical position of the foot’s lowest point. This constrain is to ensure the swing foot is always above the ground and the stance foot is always on the ground.$z_{{{\text{left}}}} (t) - z_{{{\text{right}}}} (t) < 0$, the left foot is always to the left of the right foot in the lateral direction so two legs will not interfere.

$\theta_{ft} (t) = 0$ for a stance foot during the single stance phase (foot flat).

(4) Kinetic constraints:

$F_{y} (t) > 0$, $- \mu_{x} < (F_{x} (t)/F_{y} (t)) < \mu_{x}$ and $- \mu_{z} < (F_{z} (t)/F_{y} (t)) < \mu_{z}$ for a stance foot, where $\mu_{x}$ and $\mu_{z}$ are the friction coefficient between the foot and the ground surface.

(5) Task constraint:
  $$x_{\text{an}} (T) - x_{\text{an}} (0) = VT$$

This means after a complete walking cycle, the ankle position (or any joint position) has travelled one stride length.

The optimization scheme was implemented in MATLAB using a Sequential Quadratic Programming (SQP) algorithm from the Optimization Toolbox (Gill et al. 1981). 3D whole-body gait measurement for a healthy male subject (age: 25; weight: 68.8 kg; height: 177 cm) was used to support and validate the modelling, and a detailed description of experimental procedures can be found in a previous study (Ren et al. 2005). The three input gait descriptors taken from the measurement data were: average walking speed *V* = 1.3806 ms^−1^; gait cycle period *T* = 1.08 s; and double stance duration Td = 0.16 s. The initial values for the Fourier series coefficients were set to correspond to the model standing upright and stationary. However, to obtain as many local minima as possible, these initial values were varied by uniformly distributed random numbers. Of the hundreds of predictions attempted, most have successfully converged on a different local minimum.

## Results

**Fig. 4:**
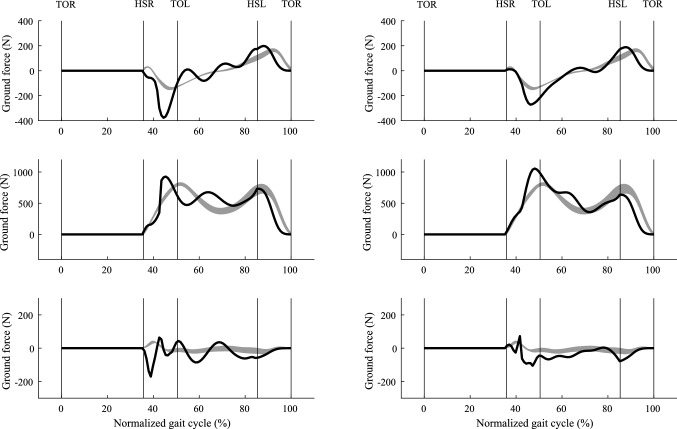
Predicted (solid line) anterior–posterior ground reaction force (top), vertical ground reaction force (middle), and lateral ground reaction force (bottom), compared with recorded force plate data (mean ± SD shaded area) from five repeated trials for one subject (age: 25; weight: 68.8 kg; height: 177 cm). The ground reaction forces on the left are from the model using performance criterion $C_{{{\text{en}}}}$ and the results on the right are from the model using criterion $C_{{{\text{tor}}}}$. The average walking speed was 1.3806 ms^−1^, and the average cycle period was 1.08 s. The swing phase is from 0 to 36%, and stance phase is from 36 to 100%. The double stance phase is from 36 to 50% and from 86 to 100%

**Fig. 5:**
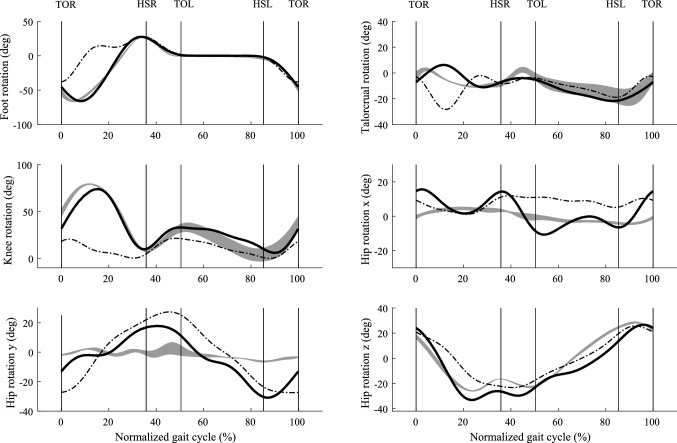
Predicted rotations of the foot, ankle, knee, and hip joints (solid line) using two performance criteria $C_{{{\text{en}}}}$ (dash-dot line) and $C_{{{\text{tor}}}}$(solid line), compared with measured data (mean ± SD shaded area) from five repeated trials for one subject (age: 25; weight: 68.8 kg; height: 177 cm). The average walking speed was 1.3806 ms^−1^, and the average cycle period was 1.08 s. The swing phase is from 0 to 36%, and stance phase is from 36 to 100%. The double stance phase is from 36 to 50% and from 86 to 100%

**Fig. 6:**
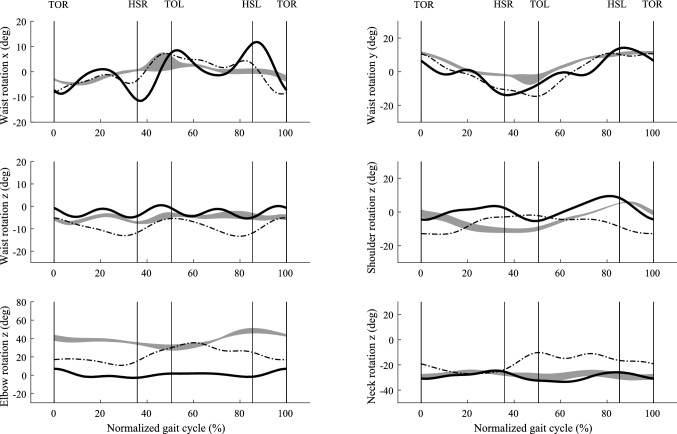
Predicted rotations of waist, shoulder, elbow and neck joints using two different performance criteria $C_{{{\text{en}}}}$(dash-dot line) and $C_{{{\text{tor}}}}$(solid line), compared with measured data (mean ± SD shaded area) from five repeated trials for one subject (age: 25; weight: 68.8 kg; height: 177 cm). The average walking speed was 1.3806 ms^−1^, and the average cycle period was 1.08 s. The swing phase is from 0 to 36%, and stance phase is from 36 to 100%. The double stance phase is from 36 to 50% and from 86 to 100%

Our predictive model successfully simulated three-dimensional whole-body walking cycle from converged solutions in an average of 37 min of CPU time on a standard laptop (Intel Core i5-6440HQ, 2.60 GHz). Values of 165 Fourier series coefficients were achieved for each simulation and over 100 converged simulations were obtained. Thereafter, we ranked the converged simulations according to the values of their objective functions and we present the simulation with the minimum value in Figs. 4, 5 and 6. Five best simulations with minimum objective function values for each criterion are shown in the Appendix (Figs. A1, A2, and A3).

**Fig. 7:**
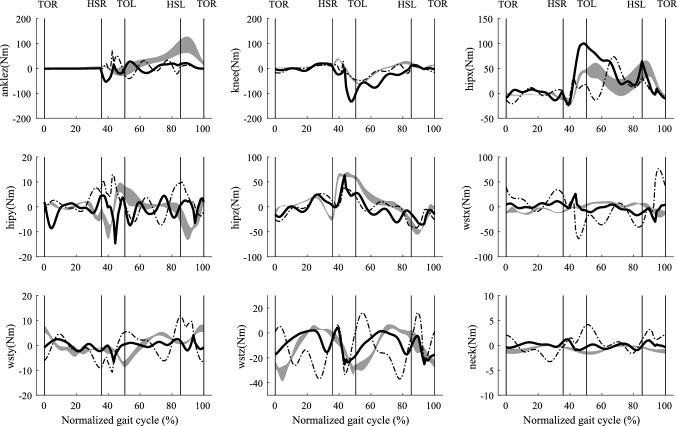
Predicted joint moments of ankle, knee, hip, waist, and neck using two different performance criteria $C_{{{\text{en}}}}$ (dash-dot line) and $C_{{{\text{tor}}}}$(solid line), compared with an inverse dynamics solution based on the measured force plate data (mean ± SD shaded area) from five repeated trials for one subject (age: 25; weight 68.8 kg; height 177 cm). The average walking speed was 1.3806 ms^−1^, and the average cycle period was 1.08 s. The swing phase is from 0 to 36%, and stance phase is from 36 to 100%. The double stance phase is from 36 to 50% and from 86 to 100%

Comparison with measurement data indicates that criterion $C_{{{\text{tor}}}}$ (sum of the time integrals of the normalized joint torques, we call it effort criterion) predicts a more realistic walking gait than criterion $C_{en}$ (mechanical energy expenditure, we call it energy criterion). Specifically, the GRFs (horizontal force $F_{x}$ and vertical force $F_{z}$) are in better agreement for effort criterion (see Fig. 4). The absolute root mean square errors (RMSEs) and relative RMSE (effort compared to energy) shown in Table 2 confirms that, for the majority of parameters, effort outperforms energy criterion. The comparison of predicted joint torque to the results from the force plate based method (Ren et al. 2008) also shows that effort criterion beats energy criterion except for the knee joint torque (relative RMSE 134.54%, see Fig. 7 and Table 2). However, energy criterion produced better predictions in the upper body joint rotation, such as waist joint rotation (relative RMSE 85.25% in left–right lateral direction) and elbow joint rotation (relative RMSE 98.59%). Although effort criterion showed good prediction in the lower limb joints and some upper body joints, it presented synchronous prediction of both shoulder rotations in flexion–extension, while in reality the arms move half a cycle out of phase with each other (see Fig. 6 and Appendix S2).

**Table 2:** RMSEs and relative RMSEs

Category	Name	RMSE of $C_{{{\text{en}}}}$	RMSE of $C_{{{\text{tor}}}}$	RMSE of $C_{{{\text{tor}}}}$ w.r.t. RMSE of $C_{{{\text{en}}}}$ (%)
GRFs (Nkg^−1^)	Fx	1.00	0.65	−35.22
	Fy	2.03	1.79	−11.93
	Fz	0.65	0.65	0.00
Joint Moments (Nmkg^−1^)	Mj_ankle	0.57	0.51	−10.63
	Mj_knee	0.21	0.49	134.54
	Mj_hip (adduction-abduction)	0.38	0.35	−5.78
	Mj_hip (internal–external)	0.11	0.09	−16.70
	Mj_hip (flexion–extension)	0.33	0.31	−4.94
	Mj_waist (left–right lateral)	0.51	0.17	−65.56
	Mj_waist (left–right)	0.10	0.05	−48.35
	Mj_waist (forward backward)	0.38	0.36	−3.62
	Mj_neck	0.03	0.01	−60.74
Joint Rotations (deg)	Foot	23.05	3.60	−84.38
	Ankle	7.46	4.02	−46.12
	Knee	30.96	7.64	−75.32
	Hip (adduction–abduction)	9.37	6.92	−26.13
	Hip (internal–external)	18.25	12.89	−29.37
	Hip (flexion–extension)	8.11	8.33	2.71
	Waist (left–right lateral)	3.11	5.76	85.25
	Waist (left–right)	5.85	6.14	5.07
	Waist (forward backward)	5.10	3.02	−40.75
	Shoulder (flexion–extension)	8.95	7.45	−16.69
	Elbow	19.26	38.24	98.59
	Neck	11.55	2.56	−77.83

Table 2 shows the absolute RMSEs in ground reaction forces (Nkg^−1^), joint moments (Nmkg^−1^) and joint rotations (deg) from energy criterion and effort criterion based prediction and the relative RMSE change between these two

The $C_{{{\text{en}}}}$ (mechanical energy expenditure) and $C_{{{\text{tor}}}}$(sum of the time integrals of the normalized joint torques) objective function values of these two simulations that we have selected were calculated and shown in Table 3. Both simulations present lower objective function values than the measurement data. To figure out how the energy and effort are shared in three dimensions during normal walking, we also calculated the components of both objective functions along *x*, *y* and *z* axis and their percentages with respect to the objective function values (Table 3). From Table 3 we find that the predictions based on effort criterion have a very similar distribution to the measurement (within 2% along all three directions), but the predictions based on energy criterion have a quite different distribution to the measurement, especially along *z* axis (68 vs 89%). These results are used to support the discussion below.

**Table 3:** Objective function values of two best simulations

	$C_{{{\text{en}}}}$		$C_{{{\text{tor}}}}$	
	Measurement (J)	Prediction (J)	Measurement (Nm/Nm)	Prediction (Nm/Nm)
Total	227.14	178.72	8.47	5.45
*Z* axis	89% (202.42)	68% (122.73)	46% (3.88)	47% (2.65)
*X* axis	9% (20.12)	21% (38.53)	38% (3.23)	39% (2.11)
*Y* axis	2% (4.60)	11% (17.46)	16% (1.36)	14% (0.69)

Table 3 shows the objective function values of two simulation that we have selected for *C*_en_ and *C*_tor_, the components along *x*, *y* and *z* axis and the percentage of these components with respective to the summation. Measurement results of *C*_en_ and *C*_tor_ were obtained by inverse dynamics driven by measured joint rotations

## Discussion

We developed a three-dimensional whole-body model to predict human walking on level ground, and then used it to explore the control strategies underpinning walking. In this study, all joint motions and ground reactions were predicted from only three simply gait descriptors: average forward velocity, gait cycle period and double stance duration. The use of inverse dynamics instead of forward dynamics has the advantage of computational efficiency because no numerical integration of the differential equations is involved, and this greatly reduced the execution time for each optimization iteration. Using quaternions to represent spatial rotations may also contribute to computational efficiency (Goldman 2009). Our model used average 37 min of CPU time on a standard laptop (Intel Core i5-6440HQ, 2.60 GHz) to converge on a solution. Although an existing simulation with similar complex model has much shorter calculation time (512 CPU seconds), it is not fair comparison since they used much faster software SNOPT for its SQP algorithm (Xiang et al. 2009).

Our three-dimensional model was informed by our previous work on a two-dimensional walking model (Ren et al. 2007). However, it is more complex than just adding one dimension to the existing model. Firstly, the joint axis orientation in space requires accurate expression since it will work with joint rotation angles to determine the special position of the next connecting segment. In order to achieve better prediction results, we optimized the orientation of the joint axes for ankle, knee, elbow and neck joint by minimizing the least square error of rotation matrix between the calculated values and the measurement data. We believe these optimal three-dimensional axis orientations, that better represent the anatomical structure, can help to improve prediction accuracy. We want to point out that joint axes from literature or OpenSim can also achieve reasonable walking gaits, although the kinematics prediction may be not as close to the measurement data as using optimized joint axes. Joint axes will affect the prediction of joint rotation but not the walking optimization (prediction) process. For example, our two-dimensional predictive model (Ren et al. 2007) still achieved reasonably good prediction, and all the joint axes were perpendicular to the sagittal plane. Therefore, the measurement data here is nice to have but not essential to have for the joint axes. Secondly, the two hip joints (represented by two ball-in-socket joints) and the waist joint (represented by one ball-in-socket joint) in the three-dimensional model has greatly increased the complexity of the model. Extra constraints in frontal and transversal plane were added, such as the foot swing (left foot is always to the left side of right foot) and the trunk rotation (the transversal rotation of trunk is constrained to keep vision straight ahead).

Mechanical energy has proved to be a good performance criterion with a two-dimensional HAT (head, arm and trunk) model (Ren et al. 2007) but it did not perform well in this three-dimensional prediction. The GRFs had stronger fluctuation and larger amplitude comparing to GRFs predicted by effort criterion. Additionally, we noticed the step width was much larger than the normal walking gait. The leg swung outwards during swing phase and it caused insufficient knee flexion (Fig. 5 and Appendix S1). In other words, the hip joint abducted to achieve floor clearance instead of bending the knee. However, in two-dimensional model, the ground clearance can only be completed by knee flexion in the sagittal plane. This demonstrates how the three-dimensional model allows a wider range of control strategies to satisfy the constraints defining walking. The large step width from this prediction based on minimizing mechanical energy indicates the optimization may need to include the walking stability objective in the frontal plane too.

Although theoretical studies using simple biped models have indicated that, in walking, energy is minimized (Srinivasan and Ruina 2006; Srinivasan 2011), mechanical energy is not seen in any existing three-dimensional gait prediction. Minimizing the joint torque has been widely used in the past (Nubar and Contini 1961; Gruver et al. 1979; Redfield and Hull 1986; Koopman et al. 1995; Xiang et al. 2009). The reason of this criterion being used less in nowadays may be that the joint moment/torque has no “scaling factor” directly related to the physiological characteristics of muscle, such as the muscle activation or the muscle stress. However, the minimization of joint torque criterion has its own advantages: first, it can predict the kinematics without building complex muscle models inside the optimization, therefore, it can reduce the computation load and increase the prediction speed; secondly, although joint torque does not directly relate to the physiology of muscles, research on the comparison of sum of the absolute values of the joint torque to metabolic energy measurements (Burdett et al. 1983) has indicated that joint moments can be used as predictors of metabolic energy consumption; thirdly, the normalization of joint torque by its maximal isometric value in each rotational direction represents the load sharing between synergistic muscles (Dul et al. 1984). Therefore, this criterion can also be referred to as a fatigue criterion (Koopman et al. 1995; Ackermann and van de Bogert 2010). Finally, the minimisation of joint torque criterion have performed well in previous researchers’ work (Marshall et al. 1989; Koopman et al. 1995; Xiang et al. 2009), for example, the comparison of prediction of segmental kinematics between different criteria has shown that minimizing joint torque produces one of the best three simulations of single stance phase of walking (Marshall et al. 1989).

Other criteria were not considered in our model, such as head stability and the foot–ground impact. The head stability criterion shows best prediction for the HAT kinematics in the simulation of single stance phase of walking (Marshall et al. 1989). But head stability by its own has limited capability in the lower limb kinematics prediction. The head stability criterion could be considered and combined with existing optimization criteria (mechanical energy or the time integral of joint torque) to help predict the motion of head and trunk. Our model for now has constrained the rotation of trunk and head by constraining the waist and head joint to a smaller range. The predicted rotation of these two joints show that they follow the measurement data well. The foot–ground impact criterion on its own has shown good prediction close to experimental data for lower limb kinematics but less good prediction in ground reaction force and joint moments (Veerkamp et al. 2021). Combining the foot–ground impact with other criteria have shown better prediction in kinematics, ground reaction forces and joint moments. But the prediction capacity of foot–ground impact criterion in the upper body, such as the torso and head motion were not shown. We understand that the three-dimensional whole-body prediction will need more than one criterion to perform well. The setup of a three-dimensional predictive model based on inverse dynamics was introduced and the independent criterion was explored first. The multiple criteria study including introducing the body stability and the trade-off between walking energy and stability into the predictive model will be the focus of our next paper.

In order to understand how the energy and effort are shared in three dimensions and why the two criteria lead to different outcomes, we calculated the components of both objective functions along *x*, *y* and *z* axis and their percentages with respect to the objective function values (Table 3). From the measurement data, we found the majority of mechanical energy was consumed along *z* axis (89%), with 9% along *x* axis and only 2% along *y* axis. This distribution explains why mechanical energy criterion leads to good gait predictions with a two-dimensional model (Ren et al. 2007). Table 3 also shows the distribution of the predictive model based on two different criteria. The effort criterion exhibits almost the same distribution of joint torque in three dimensions as the measurement: 47 vs. 46% (*z* axis), 39 vs. 38% (*x* axis), and 14 vs. 16% (*y* axis). However, the energy criterion presented a much different distribution of mechanical energy to measurement: 68 vs 89% (*z* axis), 21 vs 9% (*x* axis), and 11 vs 2% (*y* axis). We believe this distribution is achieved because, in the criterion, the joint torque about each joint axis is normalized by its expected maximum (from the literature). However, the mechanical energy expenditure criterion doesn’t involve any weighting about the three joint axes, and this may be one reason why it performed less well. This indicates the energy sharing between the three dimensions may play an important role in the human walking control. The values of objective function for those two simulations we have selected are found smaller than the values obtained from measurement data (Table 3). This indicates that a trade-off between the energy or effort criterion and other criteria (such as stability) underlies the control strategy of human walking. Other criteria may partially sacrifice the energy or effort efficiency.

Although the prediction of lower limb joints and waist joints rotations is good by using effort criterion, a limitation of this model is the upper limb joints prediction is poor. Specifically, the anterior–posterior motion of the shoulder joints is such that the two arms move in a synchronous manner, with the two arms passively swaying out of phase with the motion of trunk body. This was due to our effort criterion seeking to minimize joint torque. Therefore, a combination of objective functions may be required, possibly including minimization of the deviation of joint angles from their neutral angle positions (Kwon et al. 2017) or including whole-body stability criterion (Herr and Popovic 2008).

Our three-dimensional whole-body predictive model provides a powerful tool for understanding human locomotion. It can predict the kinematics without building complex muscle models inside the optimization, therefore, it can reduce the computation load and increase the prediction speed. Comparing to those complex muscle driven models that are based on forward dynamics method (Falisse et al. 2019), our kinematics driven model can be combined with inverse muscle models to predict muscle excitations and muscle energy consumption as well. In fact, the underlying physics is the same for forward or inverse dynamic method. We use our model to explore the underlying control strategies by comparing different performance criteria. In this paper, we compared mechanical energy expenditure with the sum of the time integrals of the normalized joint torques. Good prediction of lower limbs, trunk and the head motion can be achieved by minimizing the joint torque, but the poor prediction of upper limbs needs multiple criteria into the model. It has the potential to apply in the rehabilitation engineering if our model is extended to meet the requirement accordingly. An example of application is to assess how altered neuro-musculoskeletal properties affect gait performance by including inverse muscle models in our model to predict muscle excitations. Finally, gait cycle duration and stride length could be unknowns to be predicted during optimization, thereby enabling investigation of the velocity-stride length relationship during walking.

## Electronic supplementary material

Below is the link to the electronic supplementary material.

Supplementary file1 (EPS 113 kb)

Supplementary file2 (EPS 232 kb)

Supplementary file3 (EPS 232 kb)

Supplementary file4 (AVI 2869 kb)

Supplementary file5 (AVI 2825 kb)

Supplementary file6 (DOCX 26 kb)

## References

[CR1] Ackermann M, van de Bogert AJ (2010). Optimality principles for model-based prediction of human gait. J Biomech.

[CR2] Anderson FC, Pandy MG (2001). Dynamic optimisation of human walking. J Biomech Eng.

[CR3] Bessonnet G, Marot J, Seguin P, Sardain P (2010). Parametric-based dynamic synthesis of 3D-gait. Robotica.

[CR4] Burdett RG, Skrinar GS, Simon SR (1983). Comparison of mechanical work and metabolic consumption during normal gait. J Orthop Res.

[CR5] Cavagna GA, Kaneko M (1977). Mechanical work and efficiency in level walking and running. J Physiol.

[CR6] Chow CK, Jacobson DH (1971). An optimal programming study of human gait. Math Biosci.

[CR7] Davy DT, Audu ML (1987). A dynamic optimisation technique for predicting muscle forces in the swing phase of gait. J Orthop Res.

[CR8] de Leva P (1996). Adjustments to Zatsiorsky-Seluyanov’s segment inertia parameters. J Biomech.

[CR9] Dorn TW, Wang JM, Hicks JL, Delp SL (2015). Predictive simulation generates human adaptations during loaded and inclined walking. PLoS ONE.

[CR10] Dul J, Townsend MA, Shiavi R, Johnson GE (1984). Muscular synergism I-II. J Biomech.

[CR11] Falisse A, Serrancoli G, Dembia CL, Gillis J, Jonkers I, De Groote F (2019). Rapid predictive simulations with complex musculoskeletal models suggest that diverse healthy and pathological human gaits can emerge from similar control strategies. J R Soc Interface.

[CR12] Fregly BJ, Reinbolt JA, Rooney KL, Mitchell KH, Chmielewski TL (2007). Design of patient-specific gait modifications for knee osteoarthritis rehabilitation. IEEE Trans Biomed Eng.

[CR13] Gill PE, Murray W, Wright MH (1981). Practical optimization.

[CR14] Goldman R (2009). An integrated introduction to computer graphics and geometric modelling.

[CR15] Gonosova Z, Linduska P, Bizovska L, Svoboda Z (2018). Reliability of ankle-foot complex isokinetic strength assessment using the Isomed 2000 dynamometer. Medicina.

[CR16] Gruver WA, Ayoub MA, Muth MB (1979). A model for optimal evaluation of manual lifting tasks. J Saf Res.

[CR17] Herr H, Popovic M (2008). Angular momentum in human walking. J Exp Biol.

[CR18] Kai HK, Mombaur KD, Soueres P (2012) Studying the effect of different optimization criteria on humanoid walking motions. In: International conference on simulation, modeling, and programming for autonomous robots, pp 221–236.

[CR19] Kaminski TW, Perrin DH, Gansneder BM (1999). Eversion strength analysis of uninjured and functionally unstable ankles. J Athl Train.

[CR20] Kim HJ, Wang Q, Rahmatalla S, Swan CC, Arora JS, Malek KA, Assouline JG (2008) Dynamic motion planning of 3D human locomotion using gradient-based optimization. J Biomech Eng 130:031002(1–14). 10.1115/1.2898730 10.1115/1.2898730 18532851

[CR21] Koopman B, Grootenboer HJ, de Jongh HJ (1995). An inverse dynamics model for the analysis, reconstruction and prediction of bipedal walking. J Biomech.

[CR22] Kumar S (1996). Isolated planar trunk strengths measurement in normals: Part III—results and database. Int J Ind Ergon.

[CR23] Kwon HJ, Chung HJ, Xiang Y (2017) Human gait prediction with a high DoF upper body: a multi-objective optimization of discomfort and energy cost. Int J Hum Robot 14:1650025(1–21). 10.1142/S0219843616500250

[CR24] Lee LF, Umberger BR (2016). Generating optimal control simulations of musculoskeletal movement using OpenSim and MATLAB. PeerJ.

[CR25] Marshall RN, Wood GA, Jennings LS (1989). Performance objectives in human movement: a review and application to the stance phase of normal walking. Hum Mov Sci.

[CR26] Martin AE, Schmiedeler JP (2014). Predicting human walking gaits with a simple planar model. J Biomech.

[CR27] Martins J, da Silva JR, da Silva MRB, Bevilaqua-Grossi D (2017). Reliability and validity of the belt-stabilized handheld dynamometer in hip and knee strength tests. J Athl Train.

[CR28] Miller RH, Umberger BR, Hamill J, Caldwell GE (2012). Evaluation of the minimum energy hypothesis and other potential optimality criteria for human running. Proc Biol Sci.

[CR29] Nubar Y, Contini R (1961). A minimal principle in biomechanics. Bull Math Biol.

[CR30] Redfield R, Hull ML (1986). On the relation between joint moments and pedalling rates at constant power in bicycling. J Biomech.

[CR31] Ren L, Jones R, Howard D (2005). Dynamic analysis of load carriage biomechanics during level walking. J Biomech.

[CR32] Ren L, Jones RK, Howard D (2007). Predictive modelling of human walking over a complete gait cycle. J Biomech.

[CR33] Ren L, Jones RK, Howard D (2008). Whole body inverse dynamics over a complete gait cycle based only on measured kinematics. J Biomech.

[CR34] Saidouni T, Bessonnet G (2003). Generating globally optimized sagittal gait cycle of a biped robot. Robotica.

[CR35] Shourijeh MS, McPhee J (2014) Forward dynamic optimization of human gait simulations: a global parameterization approach. J Comput Nolinear Dyn 9:031018(1–11). 10.1115/1.4026266

[CR36] Siegler S, Liu W (1997) Inverse dynamics in human locomotion. In: Allard P et al. (eds) Three-dimensional analysis of human locomotion. Wiley, New York.

[CR37] Sreenivasa M, Millar M, Felis M, Mombaur K, Wolf SI (2017). Optimal control based stiffness identification of an ankle-foot orthosis using a predictive walking model. Front Comput Neurosci.

[CR38] Srinivasan M (2011). Fifteen observations on the structure of energy-minimizing gaits in many simple biped models. J R Soc Interface.

[CR39] Srinivasan M, Ruina A (2006). Computer optimization of a minimal biped model discovers walking and running. Nature.

[CR40] Veerkamp K, Waterval NFJ, Geijtenbeek T, Carty CP, Lloyd DG, Harlaar J, van der Krogt MM (2021) Evaluating cost function criteria in predicting healthy gait. J Biomech 123:110530(1–16) 10.1016/j.jbiomech.2021.110530 10.1016/j.jbiomech.2021.110530 34034014

[CR41] Winter DA (2005). The biomechanics and motor control of human movement.

[CR42] Xiang Y, Arora JS, Rahmatalla S, Abdel-Malek K (2009). Optimization-based dynamic human walking prediction: one step formulation. Int J Numer Meth Eng.

[CR43] Yamaguchi GT (1990). Performing whole-body simulations of gait with 3-D, dynamic musculoskeletal model. Multiple muscle systems.

[CR44] Yen V, Nagurka ML (1987) Suboptimal trajectory planning of a five-link human locomotion model. In: ASME Winter Annual Meeting on Biomechanics of Normal and Prosthetic Gait. Boston, MA, pp. 17–22.

[CR45] Zajac FE, Winters JM (1990). Modelling musculoskeletal movement systems: joint and body-segment dynamics, musculoskeletal actuation, and neuromuscular control. Multiple muscle systems.

