# Supplementary material for: A three-dimensional whole-body model to predict human walking on level ground
Source: Biomech Model Mechanobiol. 2022 Oct 26;21(6):1919–33. doi: 10.1007/s10237-022-01629-7 (PMC9700646; doi:10.1007/s10237-022-01629-7)
Supplement: Supplementary file 6 — Supplementary file6 (DOCX 26 kb) [file 10237_2022_1629_MOESM6_ESM.docx]

Fig. A1- Five best predicted (solid lines) anterior-posterior ground reaction force (top), vertical ground reaction force (middle), and lateral ground reaction force (bottom), compared with recorded force plate data (mean ± S.D. shaded area) from five repeated trials for one subject (age: 25; weight: 68.8kg; height: 177cm). The ground reaction forces on the left are from the model using performance criterion $C_{en}$ (criterion value - black line: 178.90J, red line: 183.78J, pink line: 194.54J, green line: 197.65J, blue line: 204.42J) and the results on the right are from the model using criterion$C_{tor}$(criterion value – black line: 5.45 Nm/Nm, red line: 5.45 Nm/Nm, pink line: 5.46 Nm/Nm, green line: 5.58 Nm/Nm, blue line: 5.65 Nm/Nm). The average walking speed was 1.3806ms^-1^, and the average cycle period was 1.08s. The swing phase is from 0% to 36%, and stance phase is from 36% to 100%. The double stance phase is from 36% to 50% and from 86% to 100%.

Fig. A2 – Five best predicted rotations of the foot, ankle, knee, and hip joints (solid line) using two performance criteria $C_{en}$ (solid lines in the left column) and $C_{tor}$(solid lines in the right column), compared with measured data (mean ± S.D. shaded area) from five repeated trials for one subject (age: 25; weight: 68.8kg; height: 177cm). The average walking speed was 1.3806ms^-1^, and the average cycle period was 1.08s. The swing phase is from 0% to 36%, and stance phase is from 36% to 100%. The double stance phase is from 36% to 50% and from 86% to 100%.

Fig. A3 – Five best predicted rotations of waist, shoulder, elbow and neck joints using two different performance criteria $C_{en}$( solid lines in the left column) and $C_{tor}$( solid lines in the right column), compared with measured data (mean ± S.D. shaded area) from five repeated trials for one subject (age: 25; weight: 68.8kg; height: 177cm). The average walking speed was 1.3806ms-1, and the average cycle period was 1.08s. The swing phase is from 0% to 36%, and stance phase is from 36% to 100%. The double stance phase is from 36% to 50% and from 86% to 100%.
